# Validation of a novel functional test for assessing metamorphopsia using epiretinal membranes as a model

**DOI:** 10.1038/s41598-020-71627-1

**Published:** 2020-09-10

**Authors:** Henrietta Wang, Sieu K. Khuu, Sheila Lam, Clarissa Lin, Michael Kalloniatis, Jack Phu

**Affiliations:** 1 Centre for Eye Health, University of New South Wales, Kensington, NSW Australia; 2 School of Optometry and Vision Science, University of New South Wales, Kensington, NSW Australia

**Keywords:** Retinal diseases, Eye manifestations

## Abstract

Current tests for assessing metamorphopsia do not account for confounders such as perceptual filling-in and spatial redundancy, which affect its sensitivity and repeatability. This proof-of-concept study aimed to assess the performance of a novel laboratory-based psychophysical test (Line Sag Test, LST) which addresses these issues for quantification of metamorphopsia in idiopathic epiretinal membranes. The LST quantifies perpendicular metamorphopsia at three eccentricities (3°, 6°, and 9°) along eight meridians (45° steps). Metamorphopsia was assessed using the LST and Amsler grid and the hit rates of both tests for detecting metamorphopsia were compared. Normal metamorphopsia scores using the LST did not differ significantly from 0 and fell within one step-size (p = 0.500). The LST detected significantly more cases of metamorphopsia than the Amsler grid (14/21 versus 3/21) (p = 0.003). Similarly, significantly more cases of visual distortions in asymptomatic iERMs were detected using the LST than the Amsler grid (11/18 versus 0/18) (p = 0.008). The LST has a higher hit rate compared to the Amsler grid (67% versus 14%). This work demonstrates a psychophysically-robust functional test addressing perceptual confounders is more sensitive for quantifying and localising metamorphopsia in macular disease, particularly in asymptomatic disease.

## Introduction

Metamorphopsia is one of the most common symptoms reported in macular diseases but there are limitations to the utility of some current psychophysical methods for its quantification and detection^1^. Faes et al. for example have reported poor sensitivity and repeatability in the detection of metamorphopsia associated with exudative age-related macular degeneration^2^. One such example is the Amsler grid which was the first widely adopted clinical test used to assess metamorphopsia in macular diseases^3^. The American Academy of Ophthalmology Preferred Practice Patterns guidelines for age-related macular degeneration^4^ and idiopathic epiretinal membranes^5^ recommend the use of the Amsler grid in monitoring for disease progression. While the Amsler grid offers advantages such as ease of use and portability, its major limitation is that it only provides qualitative information (i.e. metamorphopsia present or absent), thus limiting its use in the detection of functional progression. Additionally, recent studies have shown the Amsler grid to have poor sensitivity and repeatability when assessing metamorphopsia associated with macular diseases^2^. Modified versions of the Amsler grid proposed to address this issue have not been adopted into mainstream practice^6,7^. It is thought that these limitations arise from shortcomings in the design elements of the psychophysical test: duplicative elements and prolonged viewing times leave it susceptible to secondary effects from confounders such as spatial and temporal redundancy^8,9^.

The design of the Amsler grid also does not account for unstable fixation, a common finding in macular diseases^10^, which compounds the perceptual filling-in phenomenon whereby dysfunctional or missing areas of the visual field are ‘filled in’ from surrounding intact elements through neural mechanisms^11^. Other tests introduced to address the weaknesses of the Amsler grid such as the modified Amsler grid^6,7^, M-chart^12^, and preferential hyperacuity perimeter (PHP)^13^, have also been unable to completely eliminate these confounders. Therefore, there is a need for a more robust test addressing these issues to accurately detect and more objectively quantify metamorphopsia associated with macular diseases, with practical implications to guide management decisions^14^ or to facilitate the early detection of disease progression such as the conversion to neovascular age-related macular degeneration^15^.

In the present study, we describe a novel, computer-based psychophysical test, called the ‘Line Sag Test’ (LST), to objectively quantify metamorphopsia and address the abovementioned shortcomings of existing methods. A feature of this new test is the brevity of stimulus presentation (200 ms, similar to static automated perimetry), which can mitigate the effects of temporal redundancy typically arising from prolonged stimulus presentation, and reduces the time for the observer to deviate from fixation. A second key feature is the deployment of a forced-choice staircase method, allowing the objective quantification of the magnitude of distortion by the degree of line sag, instead of solely relying on subjective responses (and are therefore criterion dependent) obtained using the Amsler grid. This proof-of-concept study aimed to evaluate this new method for detecting and quantifying metamorphopsia using idiopathic epiretinal membranes (iERMs) which have historically been used as a disease model for validating tests of macular function^7,12,16^. Using this data, we also aimed to compare the performance of this test against the standard Amsler grid, the currently most widely adopted test for assessing metamorphopsia in clinical practice and one that is familiar to both practitioners and patients alike^4,5^. We hypothesise that metamorphopsia may be more accurately detected and quantified by using a psychophysical test that systematically addresses these confounders.

## Methods

### Participants

12 eyes of 12 normal participants (3 males, 9 females; mean age 60.7 ± 6.0 years) and 21 eyes of 21 participants with iERMs (11 males, 10 females, mean age 65.0 ± 11.2 years) were included in this study. All participants were presbyopic. Subjects were prospectively recruited from the Centre for Eye Health patient database. Criteria for inclusion included: the absence of any ocular pathology that would confound measures of visual function including cataracts, age-related macular degeneration and glaucoma. For the iERM cohort, membranes arising from secondary causes such as diabetic retinopathy, ocular trauma, vascular occlusions, uveitis, retinal detachments or high myopia were excluded. In addition to this, iERM subjects with macular holes were also excluded. All subjects underwent a comprehensive ophthalmic examination that included best-corrected visual acuity, Amsler grid, dilated fundus examination, colour fundus photography using the Kowa WX3D non-mydriatric retinal camera (Kowa, Japan; 45 degrees field of view), and macular (Macular Cube 512 × 128) and optic nerve head (Optic Disc Cube 200 × 200) optical coherence tomography scans obtained using the Cirrus OCT (Cirrus OCT, Carl Zeiss Meditec, Dublin, CA, USA). Visual acuity was measured using the LogMAR chart on the AT-20R Medmont Mate (Medmont International Pty Ltd, Vermont, Victoria, Australia). As part of our standardised macular assessment protocol^17^, participants were asked to indicate whether they experienced symptoms of metamorphopsia when performing activities of daily living. The specific questions pertaining to the present study were: (1) “do you experience any distortions or visual disturbances when doing near-based tasks like reading, looking at your mobile phone or in food preparation?”, (2) “do you experience any distortions or visual disturbances when looking at screens like the television or computer?” and (3) “have you noticed any visual disturbances when looking at regular objects in activities of daily living, such as when driving a car or walking around?”.

### The Amsler grid: the most widely adopted test

Amsler grid testing was performed on both cohorts with the conventional white-on-black grid under equal and bright room illumination, as per the original instructions described by Marc Amsler in 1953^3^. A + 3.00DS add correction on top of the participant’s distance prescription was used with the untested eye occluded. A viewing distance of 33 cm was used thus each small square corresponded to one visual angle degree. If the participant responded positively to perceiving visual distortions, this was classified as an Amsler grid positive result. Conversely, if the participant reported no perceivable distortion within the Amsler grid, this was defined as an Amsler grid negative result. Best-corrected distance acuity, near acuity and contrast sensitivity was also measured for both cohorts.

### Design of the line sag test (LST)

**Figure 1:**
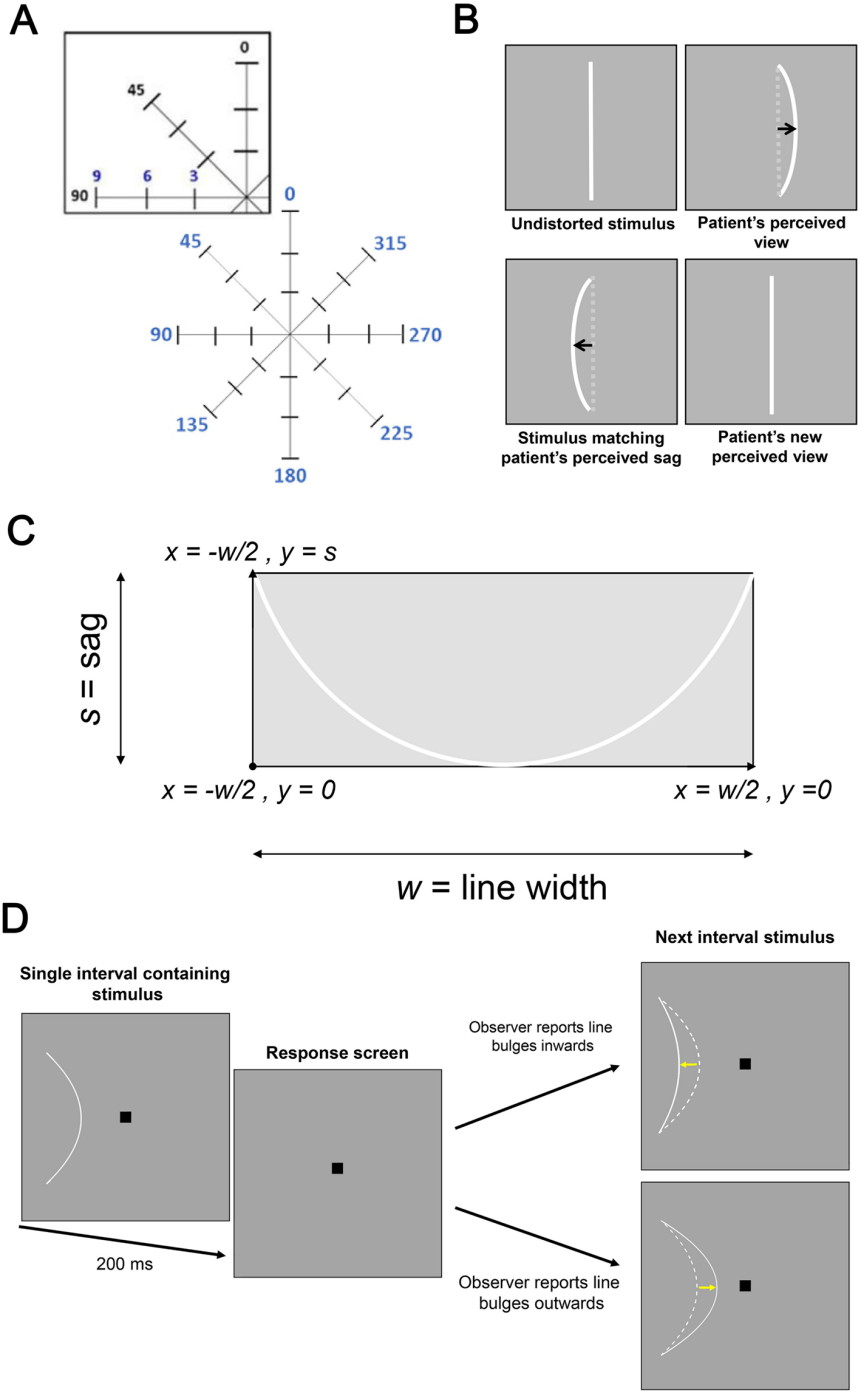
(**A**) An example of the Line Sag test which uses a staircase method with a single interval forced choice procedure. A curved line is presented briefly for 200 ms and following each presentation, the observer must indicate whether they perceived the line to be bending inwards (i.e. towards fixation) or outwards (i.e. away from fixation). The stimulus is then adjusted based on their response and the process is repeated until two reversals are achieved. (**B**) An illustrative representation of the theoretical framework underpinning the LST and its measurement of metamorphopsia. (**C**) The top right inset shows test locations at which stimuli were presented in the present study: 0° to 315° in 45° increments and at 3°, 6°, and 9°from fixation. (**D**) An illustrative example of the equation used to derive the distorted line for the LST. Equation described in the manuscript text. Figures in (**A**–**C**) were generated using Adobe Photoshop 2020 (Adobe Systems Incorporated, San Jose, CA, USA) and (**D**) was generated using GraphPad Prism version 7 (GraphPad, La Jolla, CA, USA).

The LST was designed to overcome some limitations of current functional tests. In this pilot study, we designed the LST to quantify perpendicular metamorphopsia across eight meridians (0° to 315° in 45° increments) at three eccentricities (3°, 6° and 9°) (Fig. 1A). As a significant portion of metamorphopsia in iERMs is thought to arise from displacement of the photoreceptors due to tangential traction towards the fovea^18^, the fixation point was not tested. As this was a feasibility study, we fixed the eccentricity and meridian for testing across all subjects. However, once structure–function relationships between quantification of perceptual distortion and iERM severity are better established^19^, it would be possible to further tailor the test to specific regions of interest, which may in some cases include the fovea. Similarly, the choice of a perpendicularly oriented line was also based upon the mechanism of traction associated with iERMs^20^. Perceived metamorphopsia is quantified by presenting successive stimuli with varying extents of sag, defined as the extent of maximal curvature of the stimulus from a straight line. This measure was quantified in arc minutes.The concept underlying the LST is similar to a ‘reverse Amsler grid’. When the observer is presented with a stimulus with an extent of line distortion matching their perceived degree of metamorphopsia, they will report viewing it as a straight line (Fig. 1B). The LST aims to ‘neutralise/correct’ distortion perceived by the observer by presenting a stimulus with the same magnitude of perceived metamorphopsia in the opposite direction. The extent of distortion required to neutralise the perceived metamorphopsia represents the endpoint for the test. This method of eliciting responses offers a more empirical method for quantifying metamorphopsia associated with iERMs. The curve was a parabola defined as:

$$y = \frac{{s \cdot x^{2} }}{{\left( {\frac{w}{2}} \right)^{2} }}$$

With the span of *x* ranging from − *w*/2 to *w*/2, where *w* was the width of the curve, and *s* was the sag that was varied between trials (Fig. 1C).

Stimuli were generated with a custom written software (MATLAB version 7 and Psychtoolbox version 3.0.11; MathWorks, Natick, MA) and presented on a linearised iMac 27-inch computer with a frame rate of 60 Hz. The working distance was 50 cm and a head and chin rest were used to ensure this working distance remained constant throughout testing. A trial frame with a wide aperture lens (38 mm) was used to provide accurate refractive correction. Refractive correction was calculated as a + 2.00DS addition lens on top of the subject’s distance refractive error to account for the working distance. One eye was tested with the other occluded and testing was conducted with natural pupils.

A staircase method using a single interval force choice procedure was applied to determine the metamorphopsia score (representing the magnitude of perceived distortion) at each of the 24 locations with the endpoint of the test being the threshold of sag where the observer reports no distortions (i.e. perception of a straight line). The starting extent of sag was randomised for each run. This resulted in varying test durations between subjects and between runs. Based on the step sizes (beginning with 0.5^o^, or 30 arc minutes, and halving with each reversal to a minimum step size of 0.0625^o^, or 3.75 arc minutes) and number of reversals, the minimum amount of time for stimuli presentation alone would be 3.2 arc minutes per eccentricity if the starting sag was the ground truth result (Fig. 1D). The stimulus was a white bar (with thickness of 0.05°, length of 2°, and log Weber contrast + 0.7) presented briefly (200 ms) upon a white–grey background of uniform luminance (187.5 cd m^−2^). The stimulus was oriented perpendicularly to each of the eight test meridians.

The starting meridian, starting sag at each meridian, and order of meridians tested was varied to minimise order effects. During each run, the stimulus was presented for 200 ms at one of the eight meridians and observers were required to complete a forced choice task, indicating using a button press whether the line appeared to be bulging inwards (towards fixation) or outwards (away from fixation). If unsure, the observers were asked to guess. Observers had as much time as they needed to provide a response. The sag of the line was then adjusted accordingly based on the subject’s response using a staircase procedure (to a minimum of 3.75 arc minutes increment steps, as described above) to neutralise the amount of perceived sag: an “inwards” response triggered the sag to move outwards, and vice versa for an “outwards” response. The degree of sag following the initial presentation of the starting point was varied depending on the observer’s response. For example, if the stimulus presented is an inward bulging line and the observer indicates they perceive the line to be bulging inwards, the extent of sag (i.e. the extent of deviation from a straight line) would be reduced for the successive presentations until there is a reversal whereby the observer reports the line to be bulging outwards. Conversely, if the same inward bulging stimulus is presented however the observer indicates they perceived the line to be bulging outwards, the extent of sag would be increased for the successive presentations until a reversal is reached (i.e. the observer reports the line to be bulging inwards). Each run consisted of six staircase reversals with the final four averaged to provide the sag threshold. Each run was repeated at least twice for each eccentricity thus a minimum of eight threshold measurements are obtained for each subject at each location. These measurements were then averaged to obtain an average metamorphopsia score for each location. Other tests used to identify or quantify metamorphopsia use subjective psychophysical procedures that are known in principle to have more criterion biases (e.g. Method of Adjustment used in PHP and Method of Limits used in M-Charts) compared to the forced choice staircase procedure used in the LST. The greater degree of objectivity in the LST arises from knowing the actual physical degree of sag of the presented stimulus, i.e. it is known to be inward or outward directed relative to a straight line. A forced choice procedure reduces the subjectivity of a response by forcing the observer to guess at situations of maximal uncertainty (e.g. near threshold),and the correctness of the response can be verified by comparing the observer’s response to the actual sag of the line. This limits responses such as abstaining (the yes–no response in static perimetry) and those borne from criterion bias as modelled by Signal Detection Theory^21^. We have previously illustrated the use of this psychophysical method for eliminating the phenomenon of statokinetic dissociation^22–24^.

The metamorphopsia scores for the normal cohort were used to obtain an expected normal distribution of values. Thus, positive detection of metamorphopsia using the LST in the iERM cohort was defined as a metamorphopsia scores deviating more than ± 3.08 SD from normal cohort’s average score at any location (i.e. outside of the 99.79% distribution limits) to account for a potentially increased false positive rate when sampling from 24 locations.

### Statistical analysis

All statistical analyses were performed using GraphPad Prism version 7 (GraphPad, La Jolla, CA, USA). Statistical comparisons (t-tests and Wilcoxon rank sum tests) between the groups were conducted. Though there were differences in the outcome of the normality test, application of either statistical test produced no difference in whether or not statistically significant differences existed. We report the outcome of the non-parametric statistical tests as it makes fewer assumptions about the distribution characteristics of the continuous data. The repeatability of measurements was evaluated by the repeatability coefficient (RC) using the Bland–Altman analysis^25,26^. The formula used for calculating RC specified by Bland–Altman is as follows: RC = 2.77 × S_w_ where S_w_ is the standard deviation of the difference between repeated measurements. A Fisher’s exact test was used to compare the hit rates of the Line Sag Test and the Amsler grid with *p* < 0.05 considered to be statistically significant.

### Ethical approval

This was a prospective, cross-sectional study. The study adhered to the tenets of the Declaration of Helsinki and ethics approval was provided by the Human Research Ethics Committee of the University of New South Wales. Participants provided their written informed consent prior to participation in the study.

## Results

### Demographic data and traditional measures of visual function

The characteristics of the iERM and normal cohorts are described in Table 1. There was no significant difference in age between the normal and iERM cohorts (*p* = 0.13). The difference between central foveal thickness of the iERM (319.5 ± 73.3 µm) and normal (270.0 ± 16.7 µm) cohorts was borderline significant (*p* = 0.068), indicating that the epiretinal membranes included in this study were from the milder end of the disease spectrum.The functional characteristics of both cohorts are shown in Table 2. There was no significant functional difference in the best-corrected visual acuity, the presence of Amsler grid distortions nor the presence of self-reported metamorphopsia between the two groups (*average p-value* = 0.31). No normal subjects reported metamorphopsia with the Amsler grid.

**Table 1:** Demographics and clinical characteristics of iERM and normal subjects.

	iERM (n = 21)	Normal (n = 12)	p-value
Age (years ± SD)^a^	65.0 ± 11.2	60.7 ± 6.0	0.1347
Gender (male:female, n)^b^	11:10	3:9	**0.026**
**Ethnicity**			
Caucasian	7 (33.3%)	9 (75.0%)	
East Asian	11 (52.4%)	1 (8.3%)	
Hispanic	1 (4.8%)	1 (8.3%)	
Indian or Pakistani	2 (9.5%)	1 (8.3%)	
Refractive error (diopters ± SD)^a^	− 1.67 ± 3.02	0.83 ± 1.24	**0.001**
Central foveal thickness (microns ± SD)^a^	319.5 ± 73.3	270 ± 16.7	0.068

Bolded values indicate a statistically significant result of at least p < 0.05.

*iERM* idiopathic epiretinal membrane, *SD* standard deviation.

^a^Welch’s t-test.

^b^Fischer’s exact test.

**Table 2:** Measures of visual function in the iERM and normal groups.

	iERM (n = 21)	Normal (n = 12)	p-value
BCVA (logMAR ± SD)^a^	0.08 ± 0.08	0.05 ± 0.09	0.158
Contrast sensitivity (logMAR ± SD)^a^	1.61 ± 0.01	1.68 ± 0.01	**0.019**
Self-reported perception of metamorphopsia (present:absent, n)^b^	3:19	0:12	0.169
Amsler distortions (present:absent, n)^b^	5:16	0:12	0.09
Inverse sag test (arc minutes ± SD)^a^	3.33 ± 6.13	1.29 ± 3.84	** < 0.0001**

Bolded values indicate a statistically significant result of at least p < 0.05.

*iERM* idiopathic epiretinal membrane, *BCVA* best corrected visual acuity, *SD* standard deviation.

^a^Welch’s t-test.

^b^Fischer’s exact test.

### Functional test results: the line sag test

**Figure 2:**
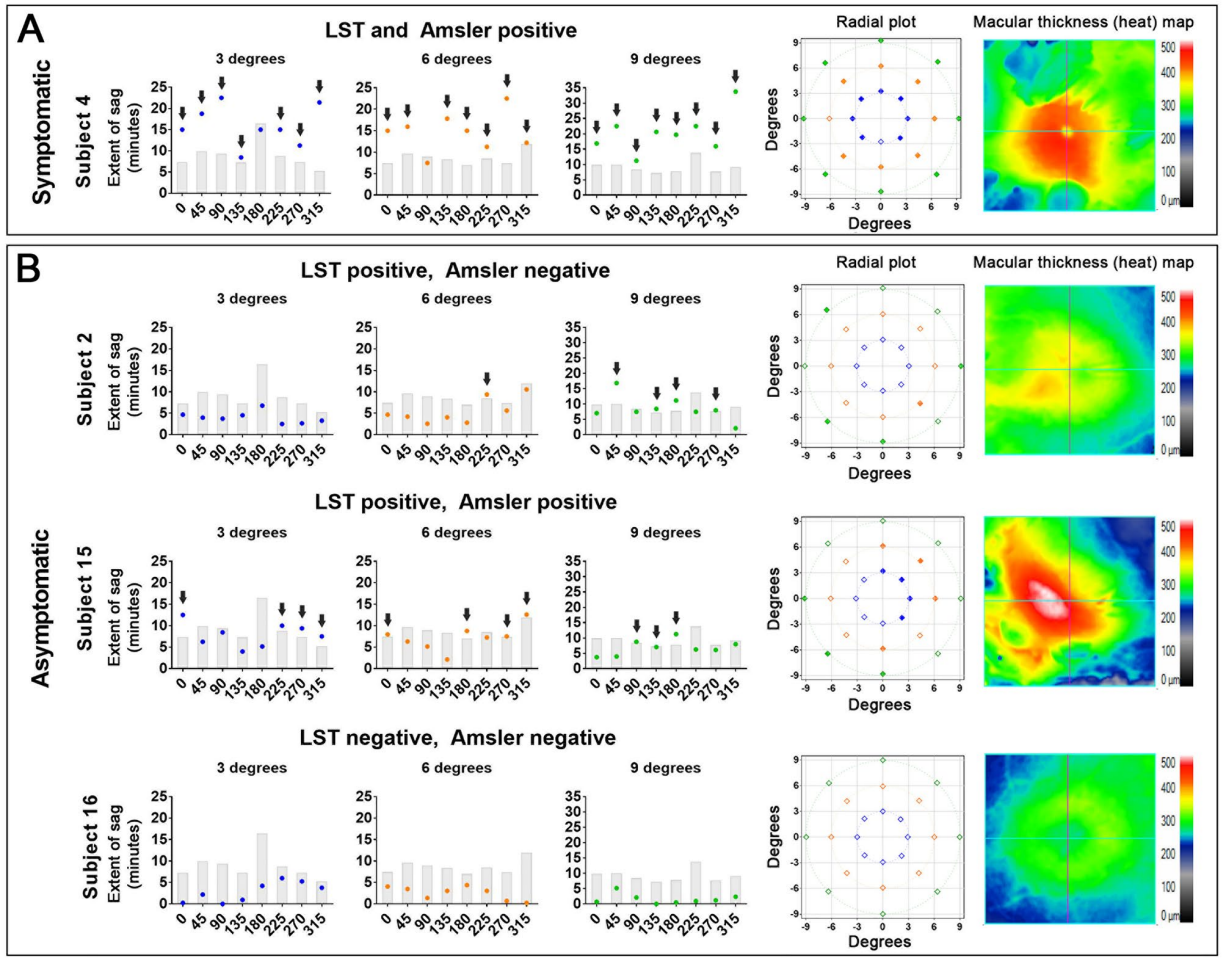
The graphs show the extent of sag threshold measured at each of the 24 locations arranged by eccentricities of 3, 6 and 9 degrees (blue, orange and green respectively) for both (**A**) symptomatic and (**B**) asymptomatic iERM subjects. For clarity and to maximise the dynamic range, sag thresholds have been converted into arc minutes for the y-axis. The grey bars indicate 2 SD magnitude of sag from the average of the normal cohort. Black arrows are used to indicate locations with metamorphopsia scores outside 2 SD of the normal cohort’s average. The radial plots on the right show the metamorphopsia scores (degrees) based on their spatial location. Locations with metamorphopsia scores outside ± 2 SD of the normal mean are shown as ‘filled-in’ points. The Cirrus HD-OCT (Cirrus OCT, Carl Zeiss Meditec, Dublin, CA, USA) macular thickness (heat) maps have been included on the right hand side of the Figure for each of the respective iERM subjects. Warmer colours (e.g. yellow or red) represent thicker retinal measurements while cooler colours (e.g. green or blue) represent normal or thinner retinal thickness measurements. Graphs and radial plots within this figure were generated using generated using GraphPad Prism version 7 (GraphPad, La Jolla, CA, USA).

The observed mean (standard deviation) test time for each run was 161.0 ± 16.3 s and 211.9 ± 51.5 s for the normal and iERM cohorts, respectively, with the latter showing a significantly longer test duration (*p* = 0.0043). Note that there was no difference in test duration between test eccentricities (*p* > 0.05) and thus we grouped all durations together for the above analysis. There was no significant correlation between central foveal thickness (CFT) and the average metamorphopsia score for the iERM cohort (r = 0.49, *p* = 0.055). Central foveal thickness showed a moderate correlation with the number of locations flagged as outside of ± 2 SD of the normal mean for the iERM cohort (slope = 0.046, r = 0.61, *p* = 0.003). Examples of the extent of sag for each of the eight meridians across the three eccentricities and the associated radial plots are shown for four representative subjects: (1) a symptomatic iERM subject with metamorphopsia detected by both the Amsler grid and LST, (2) an asymptomatic iERM subject with visual distortions detected by the LST but not the Amsler grid, (3) an asymptomatic iERM subject with metamorphopsia detected by the Amsler grid and LST, and (4) an asymptomatic iERM subject with no metamorphopsia detected by the Amsler grid and LST (Fig. 2). The macular thickness plots (heat maps) included on the right-hand side of the Figure are a qualitative representation of the iERM severity.

The normal cohort’s individual metamorphopsia scores measured with the LST did not differ significantly from 0 and fell within one step-size of zero (3.75 arc minutes) (Supplementary Table S1). The metamorphopsia scores at each of the 24 locations for all iERM subjects is shown in Supplementary Fig. S1. The average metamorphopsia scores measured using the LST were significantly higher in the iERM group (3.33 ± 6.13 arc minutes) compared to the normal cohort (1.29 ± 3.84 arc minutes) (*p* < 0.0001). The average difference between the metamorphopsia scores from run 1 and 2 was 0.210 ± 5.016 arc minutes in the normal cohort and 0.234 ± 5.226 arc minutes in the iERM cohort. The repeatability coefficient for the normal and iERM cohorts were 0.23 and 0.24 respectively. Bland–Altman plots were used to illustrate the difference in metamorphopsia scores between run 1 and 2 using the LST in both the normal and iERM cohort (Supplementary Fig. S2).

### Comparisons between the line sag test and the Amsler grid

Using the detection criteria described above in the Methods section, the LST was able to detect significantly more cases of metamorphopsia compared to the Amsler grid in the iERM cohort (14/21 versus 3/21) (*p* = 0.003) (Table 3). The hit rate of the LST was higher compared to the Amsler grid (67% versus 14%). Of note, for iERM patients without self-reported subjective visual symptoms of metamorphopsia (i.e. asymptomatic iERMs), the LST detected significantly more cases of metamorphopsia compared to the Amsler grid (11/18 versus 0/18) (*p* = 0.003) (Table 4).

**Table 3:** Agreement between the Amsler grid and the Inverse Sag test in the detection of metamorphopsia in the iERM cohort (*p* = 0.003 with McNemar’s test).

	Amsler grid positive	Amsler grid negative
Inverse sag test positive	3	11
Inverse sag test negative	0	7

**Table 4:** Detection of metamorphopsia in patients with and without self-reported symptoms of metamorphopsia using the Amsler grid and the Inverse Sag test for the iERM cohort (*p* = 0.003 with McNemar’s test).

	Amsler grid positive	Amsler grid negative	Inverse sag test positive	Inverse sag test negative
Self-reported distortions present	3	0	3	0
Self-reported distortions absent	0	18	11	7

## Discussion

In the present study, we describe a new method for measuring perceptual changes in patients that overcomes many of the limitations of existing subjective techniques, and evaluated it using the iERM disease model. Accurate measurement of metamorphopsia can provide crucial information to guide clinicians in the management of vitreomacular interface diseases^27^. Although conditions such as exudative age-related macular degeneration use techniques such as the Amsler grid for screening for the development of acute onset functional changes^28^, there is a potential clinical benefit for the quantification of visual functions in chronic or slowly progressive diseases such as iERMs. Our approach was demonstrably better at detecting metamorphopsia associated with iERMs compared to the most widely adopted clinical test, the Amsler grid. Additionally, it was also able to detect significantly more cases of visual distortion in otherwise asymptomatic patients, suggesting increased sensitivity to subclinical manifestations of metamorphopsia.

### The challenge of quantifying metamorphopsia

Although the Amsler grid remains the most widely adopted clinical test for assessing metamorphopsia, in recent years there has been a paradigm shift towards quantifying metamorphopsia^7,12^. The quantification of metamorphopsia is useful to clinicians for both understanding visual function and symptoms not reflected by traditional measures such as visual acuity which do not encompass non-central functional changes^29^. It is not uncommon for patients to report an improvement in visual symptoms following surgical intervention for iERMs without an associated improvement in visual acuity^30^. As such, the measurement of metamorphopsia to evaluate the quality of vision has become a key outcome measure of surgical success following surgical removal of iERMs^31^. The quantification of metamorphopsia may also be useful as a marker of functional progression that can be used in conjunction with structural parameters. Unlike the Amsler grid, the LST can both detect and quantify metamorphopsia. A current functional test quantifying metamorphopsia, the M-Chart, reports distortions in 0.2° steps^12^. The LST operates in smaller step sizes (0.0625°, or 3.75 arc minutes) and thus may be able to detect smaller changes in visual function not discernible with the Amsler grid, the most widely adopted test in clinical practice.

### Factors affecting the hit rate of tests of metamorphopsia

Currently, clinical tests often represent a compromise between robust psychophysical procedures and what is practical in a clinical setting. Tests which are more robust and thus useful for obtaining accurate thresholds of visual perception are time-consuming to perform or limited to a laboratory or research setting. Nonetheless, sources of variability, error and discordance in clinical tests have been attributed to the imperfect nature of the psychophysical tasks often performed in clinical practice. For example, we have shown previously that discordance between retinal structure and resultant visual perception using different perimetric techniques can be due to the nature of the psychophysical task^23,32^. Similarly, issues regarding visual attention and uncertainty have contributed to inaccuracies in threshold determination under conditions of disease^33^. Eliminating these sources of variability can produce measures that may be of higher fidelity with the underlying anatomical locus.

The increased hit rate of the LST relative to the Amsler grid can therefore be explained by the differences in test design. The majority of commercially available tests for assessing metamorphopsia such as the Amsler grid^3^, M-chart^12^ and D-chart^16^ are paper-based. The computer-based design of the LST allowed for the stimulus presentation duration to be strictly controlled. This modification allows for issues such as temporal redundancy from prolonged viewing periods to be addressed thus potentially improving the diagnostic yield of the test. Laboratory-based experiments have shown temporal redundancy to result in a preference to already existing or previously presented features from a visual scene^8,9^. As such, extended presentation time or unstable fixation can result in the “averaging out” of test elements from metamorphopsia tests.

Reduction of the stimulus duration also reduces the effects of unstable or poor fixation during testing as the time to initiate a saccade towards an unanticipated stimulus is approximately 200 ms^34^. This adjustment in conjunction with the randomised sequence of stimulus presentation helps to reduce the effects of unstable fixation on the metamorphopsia detection. Furthermore, use of a single stimulus against a plain background rather than repetitive stimuli such as those used in the Amsler grid or the D-Chart3,16, reduces potential confounding from spatial redundancy. In combination, these changes help to reduce the effect of visual adaptation thus reducing the serial dependence of perception. As such, subclinical metamorphopsia (i.e. visual distortions without subjective reported symptoms) can be detected and quantified by using a test addressing these factors.

### Limitations

There are a few limitations to this study. Firstly, we chose to use iERM as the disease model for evaluation, whilst other macular diseases may also manifest metamorphopsia symptoms, such as macular holes or exudative age-related macular degeneration^1^. As the mechanisms of underlying metamorphopsia may vary between diseases, the hit rate of the LST may vary across disease phenotypes. Additionally, central scotomas and poor visual acuity manifest in age-related macular degeneration can affect a test’s sensitivity and repeatability. As such, the utility of the LST for the detection of metamorphopsia in other macular pathologies such as age-related macular degeneration will need to be evaluated in future studies. A question raised by our results is the clinical implication of detecting subclinical metamorphopsia (i.e. measurable distortions without subjective symptoms). Although this may not necessarily create impetus for early surgical treatment, as the LST is able to detect subtle changes in perception, it may be able to discern small differences in perceptual change that may have a place in clinical monitoring of visual functions.

Another potential limitation of this study is the small sample size. A post-hoc effect size analysis of average metamorphopsia scores across locations flagged as abnormal showed a Hedges’ g of 0.99 suggesting an overall large combined effect size. However, given the smaller step sizes offered by the LST compared to other commercially available tests, it may be able to more accurately quantify visual distortions and monitor location-specific changes, with potential applications such as in post-operative visual function in iERMs that are not reflected by traditionally used parameters such as visual acuity. This requires further evaluation.

We only compared the diagnostic yield of the LST with the most widely adopted and accessible clinical test, the Amsler grid. Historically, the Amsler grid has also been the standard of choice in the preliminary validation of new metamorphopsia tests^35–38^ which remains relevant to present clinical practice due to its recommended use by the American Academy of Ophthalmology^4,5^. As such, tests for detecting quantifying metamorphopsia such as the M-chart or the PHP were not evaluated in the present study. Reported detection rate of visual distortions using these tests in iERMs range between 45.0–51.2%^35,36^ and 89.0–97.3%^37,38^ for the PHP and M-chart respectively. Given the differences in population characteristics between studies (which commonly recruit pre-operative, symptomatic patients) and our cohort, these results are not immediately comparable to the LST which has a detection rate of 67% in an asymptomatic iERM cohort. As the LST was designed to be more objective and to address the subjective limitations of current tests of metamorphopsia and visual distortions, future studies should evaluate the diagnostic yield of the LST relative to other available clinical tests. The average metamorphopsia scores for each subject were compared with central foveal thickness rather than using a spatially-driven structure–function approach. This location-specific structure–function relationship can be explored in future studies.

Finally, this was a proof-of-concept study using a laboratory-based test. We acknowledge the limitations of using a staircase method in the length of the test, not unlike clinical perimetry. However, like clinical perimetry, the implementation of Bayesian and adaptive approaches to thresholding could eventually speed up the test for practical clinical use^39^. The LST is delivered through a computer-based system that has the potential to be adapted to allow it to be performed on portable tablet or screen devices which have become increasingly popular as a tool for assessing visual function in clinical practice^40^. Further studies and refinements to the current test are required before the LST can be widely adopted in clinical practice. As expected, there was a longer test duration in the context of subjects with pathology, and again, a Bayesian approach would be useful to shorten test time^39^.

## Conclusion

We have developed and examined a novel psychophysically-robust test, the LST, which can detect and quantify metamorphopsia in patients with iERMS with greater hit rate compared to the Amsler grid, especially in evaluating asymptomatic cases of the disease. In conjunction, these results suggest that modifications to psychophysical testing procedures similar to the LST can improve the detection and quantification of metamorphopsia associated with macular diseases relative to the Amsler grid, which is the current clinical standard.

## Supplementary information

Supplementary Information

## Data Availability

The datasets generated during and/or analysed are available from the corresponding author on reasonable request.
