# Supplementary material for: Validation of a novel functional test for assessing metamorphopsia using epiretinal membranes as a model
Source: Sci Rep. 2020 Sep 10;10:14938. doi: 10.1038/s41598-020-71627-1 (PMC7484749; doi:10.1038/s41598-020-71627-1)
Supplement: Supplementary file 1 — Supplementary Information [file 41598_2020_71627_MOESM1_ESM.pdf]

Validation of a novel functional test for assessing metamorphopsia using epiretinal membranes as a model

Henrietta Wang[1,2] BOptom (Hons); Sieu Khuu[2] PhD; Sheila Lam[2] MClinOptom (Hons); Clarissa Lin[2] MClinOptom (Hons); Michael Kalloniatis[1,2] PhD; Jack Phu[1,2] PhD

[1] Centre for Eye Health, University of New South Wales, Kensington, NSW

[2] School of Optometry and Vision Science, University of New South Wales, Kensington, NSW

Number of Figures: 2

Number of Tables: 4

Number of Supplementary Figures: 2

Number of Supplementary Tables: 1

Corresponding Author: Jack Phu

Email: jphu@cfeh.com.au

SUPPLEMENTARY FIGURE CAPTIONS

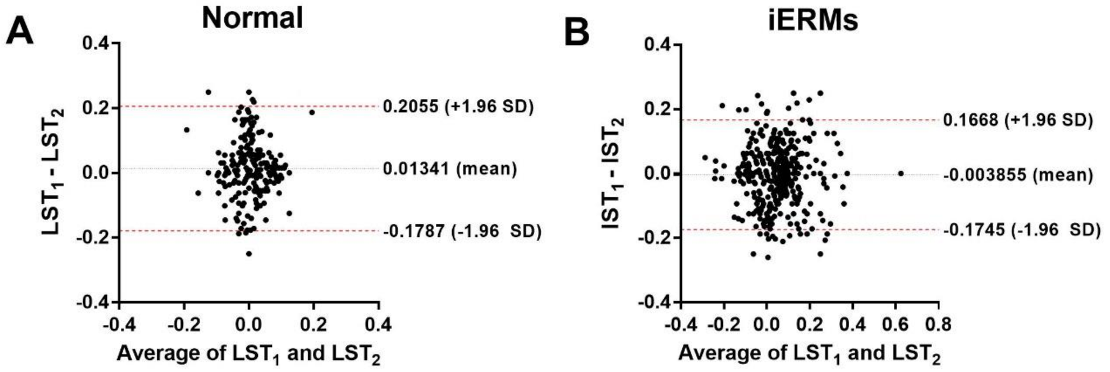

Supplementary Figure 1: Bland-Altman plots showing the repeatability of metamorphopsia scores measured using the Inverse Sag test between the two runs in the normal **(A)** and idiopathic epiretinal membrane **(B)** cohorts. The red dashed lines indicate ± 1.96 SDs above and below the mean difference between the two measurements.

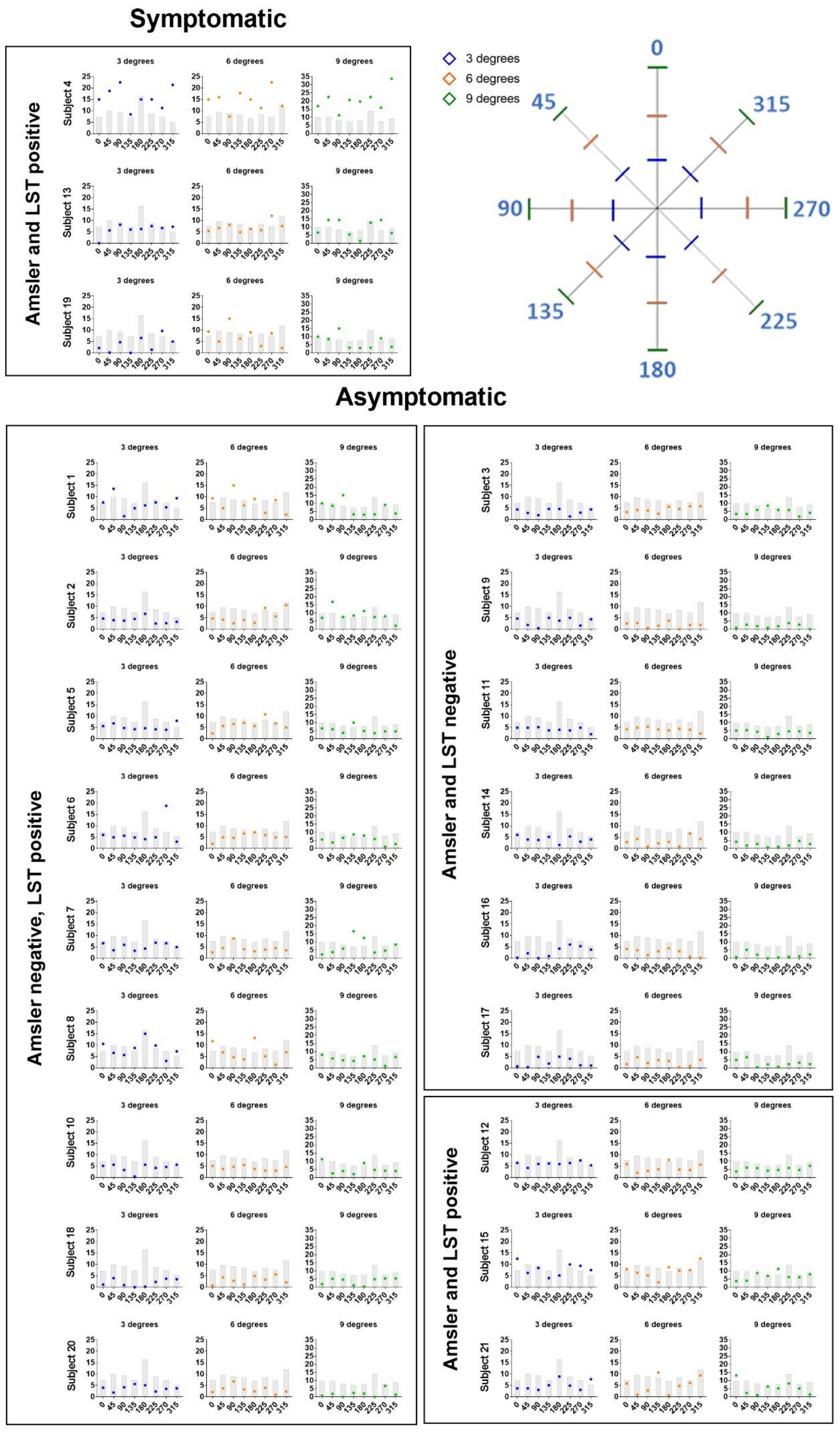
Symptomatic
Amsler and LST positive
Subject 4
Subject 13
Subject 19
3 degrees
6 degrees
9 degrees
0
45
90
135
180
225
270
315
Asymptomatic
Amsler negative, LST positive
Subject 1
Subject 2
Subject 5
Subject 6
Subject 7
Subject 8
Subject 10
Subject 18
Subject 20
Amsler and LST negative
Subject 3
Subject 9
Subject 11
Subject 14
Subject 16
Subject 17
Amsler and LST positive
Subject 12
Subject 15
Subject 21

Supplementary Figure 2: The extent of sag at each of the three eccentricities across eight meridians for all iERM subjects, grouped by their functional findings. The grey bars indicate ± 2 SD from the average of the normal cohort.

SUPPLEMENTARY TABLE

Supplementary Table 1: Sag values (mean ± SD) of the Line Sag Test for the normal population at each of the 24 locations using the staircase method.

	**3 degrees**	**6 degrees**	**9 degrees**
0	0.0186 ± 0.0686	0.0160 ± 0.0741	0.0528± 0.0573
45	0.0172 ± 0.0714	0.0510 ± 0.0810	0.0234 ± 0.0902
90	0.0337 ± 0.0672	0.0390 ± 0.0568	0.0258 ± 0.0699
135	0.0171 ± 0.1197	0.0281 ± 0.0729	0.0474 ± 0.0514
180	0.0425 ± 0.0663	0.0236 ± 0.0556	0.0254 ± 0.0587
225	0.0351 ± 0.0578	0.0468 ± 0.0484	0.0225 ± 0.1101
270	0.0331 ± 0.0681	0.0275 ± 0.0546	0.0562 ± 0.0478
315	0.0198 ± 0.0686	0.0537 ± 0.0763	0.0340 ± 0.0668
